# Access Control Model Based on Time Synchronization Trust in Wireless Sensor Networks

**DOI:** 10.3390/s18072107

**Published:** 2018-06-30

**Authors:** Zhaobin Liu, Qiang Ma, Wenzhi Liu, Victor S. Sheng, Liang Zhang, Gang Liu

**Affiliations:** 1 School of Computer Engineering, Suzhou Vocational University, Suzhou 215104, China; lwzsz@126.com (W.L.); rainbow_zhli@163.com (L.Z.); liugang@jssvc.edu.cn (G.L.); 2 School of Software, Tsinghua University, Beijing 100084, China; maq@greenorbs.com; 3 Department of Computer Science, University of Central Arkansas, Conway, AR 72035, USA; ssheng@uca.edu

**Keywords:** access control, time synchronization, wireless sensor networks, interference noise, reliability

## Abstract

Internal reliability and external safety of Wireless Sensor Networks (WSN) data transmission have become increasingly outstanding issues with the wide applications of WSN. This paper proposes a new method for access control and mitigation of interfering noise in time synchronization environments. First, a formal definition is given regarding the impact interference noise has on the clock skew and clock offset of each node. The degree of node interference behavior is estimated dynamically from the perspective of time-stamp changes caused by the interference noise. Secondly, a general access control model is proposed to resist invasion of noise interference. A prediction model is constructed using the Bayesian method for calculating the reliability of neighbor node behavior in the proposed model. Interference noise, which attacks the time synchronization, is regarded as the key factor for probability estimation of the reliability. The result of the calculations determines whether it is necessary to initiate synchronization filtering. Finally, a division of trust levels with bilinear definition is employed to lower interference noise and improve the quality of interference detection. Experimental results show that this model has advantages in system overhead, energy consumption and testing errors, compared to its counterparts. When the disturbance intensity of a WSN increases, the proposed optimized algorithm converges faster with a lower network communication load.

## 1. Introduction

Internal reliability of WSN data transmission refers to random packet losses or error packets in a wireless link caused by topological changes, dynamic links, human disturbance or packet collisions [1,2], resulting in a failure to ensure the reliability and efficiency of data transmission. External safety means some safety threats like latent invasions and attacks, including safety threats and attacks caused by passive wiretapping, data tampering and retransmission, falsification of identity, denial of service, node capture and so on, which might affect the integrity, confidentiality, authentication and serviceability of data. Therefore, it is necessary to implement access control that can protect the network from outside interference. On the other hand, time synchronization is the precondition to realize network functions like cooperative awareness, communication, energy and access control management of nodes. For instance, the algorithms like malicious nodes perceiving, topology discovery and target tracing need nodes to mark time stamp on the awareness events and information data.

The fusion of time synchronization and accurate access control remains a challenging job due to some new characteristics of WSN such as large-scale remote deployment, application of low cost hardware facilities and limit on network energy supply. There are mainly three factors that interaction between time synchronization precision and access control.

First, clock drift exists in the current hardware clock, the frequency is easy to be affected by environment and interference noise (This includes internal and external interference) [3].

Second, synchronization method based on the reduction of the interference noise relies on access control to exchange the synchronization information, uncertain delay may occur in the process of sending and receipt information of access control.

Third, the access control is one of the key artifices which affect the accuracy of time synchronization. The timestamp change caused by interference noise selects different control strategies for the node resource access behavior.

Regarding internal reliability issue, Internal interference usually sends error time information to other nodes through compromised nodes to interfere with time synchronization, such as Delay Attack. A dynamic trust model based on multiple factors was proposed [4,5,6,7]. The model improves the reliability degree between WSN nodes and ensures the safety of node data transmission through a multi-angle reliability model which combines communication, data and energy. A hybrid trust computation scheme also was proposed, which first obtains a single trust value in a whole group [8] then, within each group, all sensor nodes calculate individual trust values for all group members and group heads aggregate these trust values and forward them to a base station. Following that, the base station periodically multicasts the current state of each group to all group heads. The information encryption access technology can guarantee the integrity and confidentiality of the information packet and can effectively resist external attacks but it cannot guarantee time accuracy [9]. The Fault Tolerant Time Synchronization Protocol (FTTSP) judges and detects the internal noise of the node based on the difference between the estimated value of the transmission time and the actual value by with fault-tolerance mechanism [10]. However, the above method does not correct the time rate, the synchronization accuracy is low, the synchronization error increases rapidly and there is no abnormality in the detection time rate and the abnormality of the time rate will affect the node time running, which will increase the time error between the nodes and eventually lead to time synchronization failure.

Regarding external safety issue, External interference usually causes time synchronization failures by tampering with the communication process between two trusted nodes, such as Masquerade Attack. The Accountable and Privacy-Enhanced Access Control in Wireless Sensor Networks (APAC) solution based on group signature provides protection privacy for access control in wireless sensor networks [11]. This solution divides users in a network into groups and users in different groups are given different access authorities. This limits the query behaviors of users. Query commands of users can only pass the verification of the node when they are set according to the access authority of the group to which they belong. Han [12] analyzed various applications of the reliability model and categorized the reliability models to defend against hostile attacks.

The reliability relationships between nodes are dynamic and uncertain in a complex WSN [13,14,15] however, which do not remain the same as the relationships that are recognized when the network is established [16]. Some entities which are reliable before might become unreliable for certain reasons as time passes. The hostile behaviors of unreliable entities might threaten the normal operations of a system if changes are ignored. Additionally, due to the importance of time synchronization in WSN data transmission, time synchronization access control has become a general approach to test whether a WSN experienced a hostile attack. In addition, the above model only allows one node to transmit a single data packet in one cycle. This results in a longer end-to-end transmission delay of the data packet. As the load increases, the data packet is likely to be accumulated in the node backlog. Node buffer overflows severely affect the performance of the network.

Currently, a Bayesian method is used to study WSN access control through defining time synchronization states of nodes and the spatial correlation between time synchronizations of different nodes [17]. A clustering algorithm was proposed for tracking targets [18]. A heterogeneous event access control method was proposed based on considering the heterogeneity in the access control of WSN [19]. Meanwhile, sensor node information also has a property of time correlation, which is used to conduct relevant research on realizing access control through taking advantage of characteristics of time and spatial information of nodes [20]. Among time synchronization correlations, different distances between nodes might have a different impact on the time synchronization. Normalized treatments on the distances between nodes are conducted to quantify the impact of distances, which further optimizes the algorithm of access control [21]. Affected by the topological location of nodes, perception similarity and time synchronization information reliability of different nodes might vary.

Taking the time correlation of WSN nodes and the shortage of WSN resources into consideration, adopting the principle of light weight, this paper puts forward a method to estimate the reliability degree between nodes based on a time synchronization access control model through analyzing the feasibility degree of disturbance noise and the time synchronization of nodes.

## 2. Clock Model

Given a topological structured undirected graph G=(V,E), where V={1,2,…,n} are the nodes in the network G, and E are the communication links of all nodes, a neighbor set of node i can be defined as $N_{i}=\left\{j|(i,j)\in E,\;\forall j\in V\right\}$. Regarding node $i\in V,\;\deg(i)=\left|N_{i}\right|$, is the degree of node i. Hardware clock function [22,23] of each node is defined as follows:

(1) $$H_{i}\left(t\right)=\alpha_{i}t+\beta_{i},\;i\in V$$

where $\alpha_{i}$ is the hardware clock skew, representing clock speed and $\beta_{i}$ is the hardware clock offset [24], $\forall i\neq j,\;\alpha_{i}\neq \alpha_{j},\beta_{i}\neq \beta_{j}$. According to Formula (1), the relative logic clock relation equation of node i and j is defined as follows:

When there is no interference noise, the relation equation is defined as follows:

(2) $$H_{i}\left(t\right)=a_{ij}H_{i}\left(t\right)+b_{ij}=a_{ij}\alpha_{i}t+a_{ij}\beta_{i}+b_{ij}$$

When there is interference noise, the relation equation is defined as follows:

(3) $$H_{i}^{+}\left(t\right)=a_{ij}H_{i}\left(t\right)+b_{ij}+Q_{i}t=a_{ij}\alpha_{i}t+a_{ij}\beta_{i}+b_{ij}+Q_{i}t={\tilde{x}}_{i}t+{\tilde{y}}_{i}+Q_{i}t$$

where ${\tilde{x}}_{i}=a_{ij}\alpha_{i}$, ${\tilde{y}}_{i}=a_{ij}\beta_{i}+b_{ij}$ and $a_{ij}$ and $b_{ij}$ respectively represent the relative clock skew and the relative clock offset of node i and j [25]; ${\tilde{x}}_{i}$ and ${\tilde{y}}_{i}$ represent the clock skew and offset of node i and j caused by internal disturbance noise, such as mutual communication delay [26], measurement deviation, clock vibration and internal covert attacks [27,28]. $Q_{i}(t)\in \left[\psi_{1},\psi_{2}\right]$ defines external disturbance noise for node i from its neighbor nodes. It is a random delay from an external attack. $\left|H_{i}^{+}\left(t\right)-H_{i}\left(t\right)\right|$ is used to determine whether the node has received external interference and, if $\left|H_{i}^{+}\left(t\right)-H_{i}\left(t\right)\right|>0$, it means this node is delayed or if $\left|H_{i}^{+}\left(t\right)-H_{i}\left(t\right)\right|\notin \left[\psi_{1},\psi_{2}\right]$, this synchronization information is not trusted. When node i receives internal hidden interference, its current time $H_{i}^{+}\left(t\right)$ is not affected. However, ${\tilde{x}}_{i}$ and ${\tilde{y}}_{i}$ have deviated, for ${\tilde{x}}_{i}^{+}={\tilde{x}}_{i}+\xi$, ${\tilde{y}}_{i}^{+}={\tilde{y}}_{i}+\zeta$, the $\Delta {\tilde{x}}_{i}=\left|{\tilde{x}}_{i}^{+}-{\tilde{x}}_{i}\right|$ and $\Delta {\tilde{y}}_{i}=\left|{\tilde{y}}_{i}^{+}-{\tilde{y}}_{i}\right|$ are used as criteria to check whether the clock skew and offset of the node is normal. When $\left|{\tilde{x}}_{i}^{+}-{\tilde{x}}_{i}\right|>0$ and $\left|{\tilde{y}}_{i}^{+}-{\tilde{y}}_{i}\right|>0$, it means that time synchronization of this node is disturbed by the internal hidden interference and the synchronization information is not trusted.

## 3. Noise Detection

Assumption 1: Given a constant δ>0, if there exits an  ε (0<ε≤1)*,* for any i∈V, one can have:

(4) $$P\left\{Q_{i}\left(t\right)\in \left[\psi -\delta ,\psi +\delta \right]\forall \psi \in \left[\psi_{1},\psi_{2}\right]\right\}\geq \varepsilon$$

Generally, disturbance noise is a random variation which obeys specific distribution, such as Gaussian distribution and index distribution [29], or distributions which have a fixed mean value and variance [30] for example. High accuracy and full-time synchronization can be realized in the sense of expectation for the above specific situation. However, the noise mode of disturbance noise might have different distributions in different times and have no fixed mean value or variance, which results in failure for an existing algorithm to realize synchronization. Moreover, the hostile behavior received by node i from its neighbor node might threaten the normal operation of a system [31].

Assumption 2: The domain of a random algorithm is M∈Θ, which meets different interference $\left({\tilde{x}}_{i},\xi \right)$ and $\left({\tilde{y}}_{i},\zeta \right)$ for A⊆Range(M) and all the data packets C, $C^{\prime }\in \Theta$, with $\left\|C-C^{\prime }\right\|\leq 1$:

(5) $$P\left\{M\left(j\right)\in A\right\}\leq exp\left({\tilde{x}}_{i}+{\tilde{y}}_{i}\right)\cdot P\left\{M\left(i\right)\in A\right\}+max\left(\xi ,\zeta \right)$$

where M is an algorithm for time synchronization and access control, ${\left\|C-C^{\prime }\right\|}_{1}$ is $l_{1}$ norm distance. When ξ=0∧ζ=0 is set up, M meets the difference interference of $\left({\tilde{x}}_{i},\xi \right)$ and $\left({\tilde{y}}_{i},\zeta \right)$ under the condition of $Q_{i}(t)\in \left[\psi_{1},\psi_{2}\right]$, in which only one element’s difference is between data packet C and $C^{\prime }$. The authors refer to the two data packets whose difference is one element at most to the neighbor data packet. This can also be regarded as the disturbance protection level offered by the proposed algorithm. The smaller the parameter $\left({\tilde{x}}_{i}+{\tilde{y}}_{i}\right)$, the higher the protection level is. When the parameter is zero, it can be regarded that the time synchronization algorithm has the same distribution on the two data packets with a single data difference for any input to query the output results. These results cannot reflect any useful information on the data packet. The larger the value of $\left({\tilde{x}}_{i}+{\tilde{y}}_{i}\right)$, the more noise might be needed. This might lower the utility of data. Thus, the value needs to reach a certain balance between the disturbance protection level and the data utility.

When nodes are attacked by interference noise, the ${\tilde{x}}_{i}$ and ${\tilde{y}}_{i}$ values are unknown but according to the exchange of information between the nodes, one can get the estimated value of this moment, though this value can be out of the state at this moment. 

**Figure 1:**
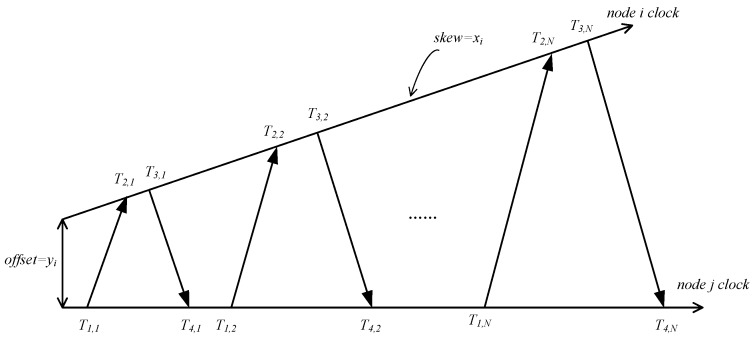
Two-way synchronous information exchange between two nodes.

The node i is taken as a reference node and the node j is a node to be synchronized. It needs to synchronize with the node i, shown in Figure 1. Assuming time synchronization information exchange happens N times, in the $k^{st}$ information exchange, node i sends synchronization information of $t_{1,k}$ at the time of $t_{1,k}$ to node j, node j receives time synchronization information ${\left[{\tilde{x}}_{i}\left(t_{1,k}\right)\;{\tilde{y}}_{i}\left(t_{1,k}\right)\right]}^{t_{1,k}}$ at $t_{2,k}$ moment and responds with the time synchronization information data packet to node i at $t_{3,k}$ moment which includes ${\left[{\tilde{x}}_{j}\left(t_{2,k}\right)\;{\tilde{y}}_{j}\left(t_{2,k}\right)\right]}^{t_{2,k}}$ and ${\left[{\tilde{x}}_{j}\left(t_{3,k}\right)\;{\tilde{y}}_{j}\left(t_{3,k}\right)\right]}^{t_{3,k}}$ and, finally, node i receives the data packet at $t_{4,k}$ moment. Here $t_{1,k}$ and $t_{4,k}$ are the local time of node i, while $t_{2,k}$ and $t_{3,k}$ are the local time of node j. Following N rounds of exchanges completed, node i acquires a series of time synchronization information data packets $\{{\left[{\tilde{x}}_{i}\left(t_{1,k}\right)\;{\tilde{y}}_{i}\left(t_{1,k}\right)\right]}^{t_{1,k}},{\left[{\tilde{x}}_{j}\left(t_{2,k}\right)\;{\tilde{y}}_{j}\left(t_{2,k}\right)\right]}^{t_{2,k}},[{\tilde{x}}_{j}\left(t_{3,k}\right)$${{{\tilde{y}}_{j}\left(t_{3,k}\right)]}^{t_{3,k}},{\left[{\tilde{x}}_{i}\left(t_{4,k}\right)\;{\tilde{y}}_{i}\left(t_{4,k}\right)\right]}^{t_{4,k}}\}}_{k=1}^{N}$ with a time stamp ${\left\{t_{1,k},t_{2,k},t_{3,k},t_{4,k}\right\}}_{k=1}^{N}$ caused by disturbance noise. A math model of the above process can be described mathematically as follows:

(6) $$t_{2,k}={\tilde{x}}_{i}\left(t_{1,k}+\upsilon +\chi \right)+{\tilde{y}}_{i}$$

(7) $$t_{3,k}={\tilde{x}}_{i}\left(t_{4,k}-\upsilon -\chi \right)+{\tilde{y}}_{i}$$

where mathematical symbol υ represents fixed delay of a node information exchange [32] and it can be considered that it is known during the synchronization period. χ represents the random delay of the mutual transmission between node i and node j, where χ obeys the Gaussian distribution of Assumption 1 [33]. Thus, there are only two unknown numbers ${\tilde{x}}_{i}$ and ${\tilde{y}}_{i}$ in Formulas (6) and (7), which can be transformed as follows:

(8) $$\{\begin{array}{c} \frac{t_{2,k}}{t_{1,k}+\upsilon +\chi }={\tilde{x}}_{i}+\frac{{\tilde{y}}_{i}}{t_{1,k}+\upsilon +\chi }\\ \frac{t_{3,k}}{t_{4,k}-\upsilon -\chi }={\tilde{x}}_{i}+\frac{{\tilde{y}}_{i}}{t_{4,k}-\upsilon -\chi } \end{array}$$

One can further change the above formula into matrices as follows:

(9) $$\left[\begin{array}{c} \frac{t_{2,k}}{t_{1,k}+\upsilon +\chi }\\ \frac{t_{3,k}}{t_{4,k}-\upsilon -\chi } \end{array}\right]=\left[\begin{array}{cc} 1 & \frac{1}{t_{1,k}+\upsilon +\chi }\\ 1 & \frac{1}{t_{4,k}-\upsilon -\chi } \end{array}\right]\left[\begin{array}{c} {\tilde{x}}_{i}\\ {\tilde{y}}_{i} \end{array}\right]$$

The established state equation of time synchronization is referred to as the state of the $k^{st}$ moment and the state measurement value of the moment can be acquired through information exchanges between two nodes. Therefore, one can estimate the state of the system by a certain method and adjust the logical clock of node i to reach a synchronization between node i and j. However, this synchronization system is not stable due to the poor anti-interference ability and easy noise disruption. 

The estimation of $Q_{i}(t)$ can be obtained by the Least Square Estimate [34], namely:

(10) $${\hat{H}}_{i}\left(t\right)=\hat{a}H_{i}\left(t\right)+\hat{b}$$

where k is the synchronization time:

(11) $$\{\begin{array}{l} \hat{a}=\frac{(k-1)\sum_{m=1}^{k-1}\left[H_{i}\left(t_{m}\right)H_{j}\left(t_{m}\right)\right]-\sum_{m=1}^{k-1}H_{i}\left(t_{m}\right)\sum_{m=1}^{k-1}H_{j}\left(t_{m}\right)}{(k-1)\sum_{m=1}^{k-1}{\left(H_{i}\left(t_{m}\right)\right)}^{2}-{\left(\sum_{m=1}^{k-1}H_{i}\left(t_{m}\right)\right)}^{2}}\\ \hat{b}=\frac{\sum_{m=1}^{k-1}H_{j}\left(t_{m}\right)}{k-1}-\hat{a}\frac{\sum_{m=1}^{k-1}H_{i}\left(t_{m}\right)}{k-1} \end{array}$$

Let $\Delta H_{i}\left(t\right)=\left|H_{i}^{+}\left(t\right)-{\hat{H}}_{i}\left(t\right)\right|$ be established and draw $\Delta H_{i}\left(t\right)\approx Q_{i}\left(t\right)$. Only if $Q_{i}(t)\in \left[\psi_{1},\psi_{2}\right]$ and ${\tilde{x}}_{i}<\xi \wedge {\tilde{y}}_{i}<\zeta$, is this synchronization information valid, otherwise it is discarded. When interference synchronization information of this node is continuously received, this node is deleted from the neighbor list and an alarm is issued. Only if $Q_{i}\left(t\right)$, can ${\tilde{x}}_{i}$ and ${\tilde{y}}_{i}$ be used as access control objects at the same time to avoid missed detection. Thus, it is necessary to find a solution for the above issues, which is why access control is needed to be introduced to the above-mentioned time synchronization model to reduce the impact from disturbance noise and to improve the reliability and the safety of data transmission through a certain control model to be described in the following section.

## 4. Access Control Model Based on Time Synchronization Trust

### 4.1. Time Synchronization Trust Relationship

Regarding the access control of sensor networks, time synchronization association refers to the perception information of node i which is, to some extent, associated to its neighbor nodes. The nearer the distance, the stronger the association and the more similar are the observed data. Therefore, it is very important whether the disturbance event test of the neighbor node is accurate. The association of sampling data between node i and its neighbor node $N_{i}=\left\{j|(i,j)\in E,\;\forall j\in V\right\}$ can be reflected by ${\tilde{x}}_{i}$ and ${\tilde{y}}_{i}$ parameters. The larger the parameter values, the more noise is in the disturbance event test of node i, caused by sampling data of the neighbor node and vice versa. 

Based on the above thought, the safety and reliability of network node access can be improved by taking advantage of the feasibility of neighbor nodes, regarding ${\tilde{x}}_{i}$ and ${\tilde{y}}_{i}$ as a reliable approach to measure the environment of neighbor nodes. Concerning node i, the confidence weight value $R_{j}$ of the perception information for the decision of neighbor node $N_{i}=\left\{j|(i,j)\in E,\;\forall j\in V\right\}$ is defined as follows [35]:

(12) $$R_{j}=\frac{1/\left({\tilde{x}}_{j}+{\tilde{y}}_{j}\right)}{\sum_{j\in N_{i}}^{deg\left(i\right)}1/\left({\tilde{x}}_{j}+{\tilde{y}}_{j}\right)}$$

where the weighted summation of all neighbor nodes is 1. The confidence weight value of node i for neighbor node $N_{i}$ is synthesized as:

(13) $$\Delta_{i\;j}=\sum_{j\in N_{i}}^{deg\left(i\right)}\left(e^{Q_{j}\left(t\right)}R_{j}\right)$$

which ensures the validity of access control under bounded noisy clock synchronization. During the process of the event test under the node access control, the confidence degree of a node is not only from reliable perception information of itself but, also, from judgement of the perception information for the external safety of its neighbor node. The subjective judgement whether $\Delta_{i\;j}$ of the node could offer accurate perception information for other nodes is largely subject to the final judgement whether there are events happening in all the neighbor nodes. When t→∞, it can realize the disturbance event test and efficiently eliminate the effects from bad nodes.

### 4.2. Access Control Model

Improve the subjective probability model by utilizing Assumption 2—the conditional probability by which node i is judged to be in convergence according to perception information of the neighbor nodes.

(14) $$P\left\{\underset{k\to \infty }{\lim}\left({\tilde{x}}_{i}\left(k\right)-{\tilde{x}}_{j}\left(k\right)\right)=0\right\}=1,\;P\left\{\underset{k\to \infty }{\lim}\left({\tilde{y}}_{i}\left(k\right)-{\tilde{y}}_{j}\left(k\right)\right)=0\right\}=1$$

This is also the node error tolerant model of access control. k is the iteration cycle of node i. Combined with formula (14), on the basis of the error tolerant model of node perception reliability based on time synchronization, suppose the node error rate is ϑ, the number of neighbor nodes with no disturbance events is γ, the probability of nodes in which disturbance events might occur is $\varsigma =\left(\left|N_{i}-\gamma \right|\right)/N_{i}$. The data of γ nodes in the neighbor node is w, which is consistent with node i. The subjective reliability probability of node i for its neighbor node $j\in N_{i}$ can be acquired through a Bayesian method [36]:

(15) $$P_{i}\left(k\right)=P_{i}\left\{\Delta {\tilde{x}}_{i}\left(k\right),\Delta {\tilde{y}}_{i}\left(k\right),E_{i}\left(w,\gamma \right)\right\}=\frac{\left(1-\vartheta \right)\gamma \Delta_{ij}}{\left(1-\vartheta \right)\gamma \Delta_{ij}+\vartheta \left(\left|N_{i}\right|-\gamma \Delta_{ij}\right)}$$

Evidently, there exists $0\leq P_{i}\left(k\right)\leq 1$ which is a kind of reliability degree test. As a result, the conformance vector of k iteration time synchronization acquired from the neighbor node $N_{i}=\left\{j|(i,j)\in E,\;\forall j\in V\right\}$ of node i is:

(16) $$P\left(k\right)=\left[P_{1}\left(k\right),P_{2}\left(k\right),\ldots ,P_{deg(i)}\left(k\right)\right]$$

Regarding node i, the behavior reliability of itself can be represented by a time series measured by itself. Regarding the periodic readout of the node as a sequence in chronological order, this readout sequence is virtually a sample value of node behavior process. Considering the limited calculation and storage capacity of the sensor, only the sample values in a certain period are kept. To improve the degree of reliability, taking the mean value ${\overset{⌣}{P}}_{i}$ of the comprehensive time synchronization conformance estimation can be defined as follows:

(17) $${\overset{⌣}{P}}_{i}\left(k\right)=\frac{1}{k}\sum_{j=1}^{deg\left(i\right)}P_{j}\left(k\right)$$

where the fluctuation of the sequence is little, that means the disturbance noise from its neighbor node $N_{i}$ to node i is little and reliability is high. Therefore, the reliability of node i can be defined intuitively as the expectation and the variance of the time synchronization conformance estimate as follows:

(18) $$E\left[P_{i}\left(k\right)\right]=\eta ,\;\rho_{i}^{2}\left(k\right)=\frac{1}{k}{\sum_{i=1}^{k}\left[{\overset{⌣}{P}}_{i}\left(k\right)-P_{i}\left(k\right)\right]}^{2}$$

Formula (18) can be used to calculate the reliability property value of a node behavior. Using the reliability calculation, the sensor node which has a higher reliability degree should be the nodes with higher time synchronization conformance and higher reliability, for example a larger $P_{i}\left(k\right)$ value and a smaller $\rho_{i}^{2}\left(k\right)$ value. Regarding node i, a larger $P_{i}\left(k\right)$ value does not mean that the $\rho_{i}^{2}\left(k\right)$ value is large or small and vice versa. Thus, mapping $f\left[{\overset{⌣}{P}}_{i}\left(k\right),\rho_{i}^{2}\left(k\right)\right]$ is introduced to ensure the node behavior reliability degree has a positive correlation with ${\overset{⌣}{P}}_{i}\left(k\right)$ and a negative correlation with $\rho_{i}^{2}\left(k\right)$. The node behavior reliability degree is defined by bilinearity:

(19) $$U_{i}\left(k\right)=f\left[{\overset{⌣}{P}}_{i}\left(k\right),\rho_{i}^{2}\left(k\right)\right]=\left[1-\varepsilon \rho_{i}\left(k\right)\right]{\overset{⌣}{P}}_{i}\left(k\right)$$

Thus, the reliability degrees of node i for neighbor node j can be lined up according to $U_{i}\left(k\right)$ value and then graded by different thresholds $\phi \in \left[\phi_{1},\phi_{2}\right]$.

### 4.3. Algorithm Design

**Figure 2:**
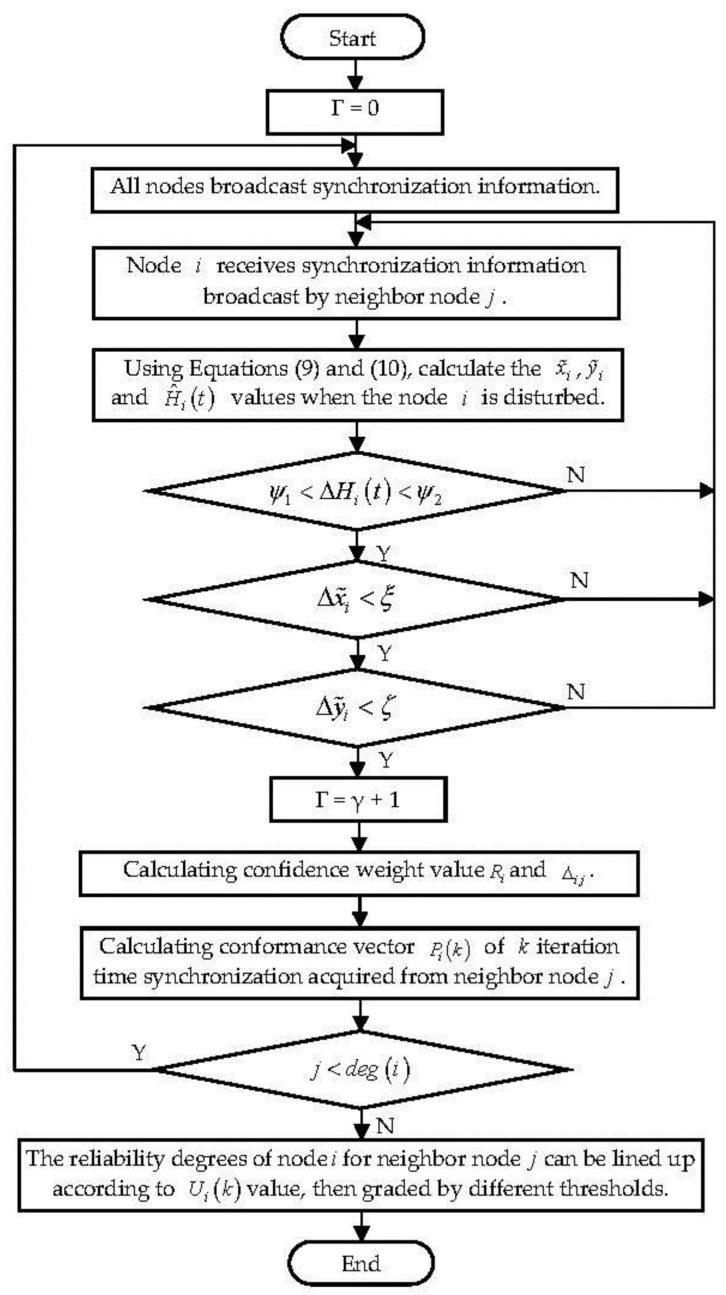
The basic steps of CEACM-TS.

The detailed steps of the authors’ algorithm, as showed shown in Figure 2, Consistency Estimates of Access Control Model based on Time Synchronization (CEACM-TS), are as follows:

Step 1: Number of neighbor nodes with no disturbance events is *γ*, the initial value of *γ* is 0.

Step 2: The node broadcasts the synchronization information and receives the synchronization information sent by its neighbor nodes.

Step 3: Node i receives synchronization information broadcast by neighbor node *j*. The node calculates the clock skew and clock offset caused by the interference noise received by the hidden attack inside the node according to Formula (9), respectively. The estimation of $Q_{i}\left(t\right)$ can be obtained by Least Square Estimate, namely $Q_{i}\left(t\right)\approx \Delta H_{i}\left(t\right)=\left|H_{i}^{+}\left(t\right)-{\hat{H}}_{i}\left(t\right)\right|$.

Step 4: Only if $Q_{i}(t)\in \left[\psi_{1},\psi_{2}\right]$ and ${\tilde{x}}_{i}<\xi \wedge {\tilde{y}}_{i}<\zeta$ is this synchronization information valid, otherwise it is discarded. $Q_{i}\left(t\right)$, ${\tilde{x}}_{i}$ and ${\tilde{y}}_{i}$ can be used as access control objects at the same time to avoid missed detection. 

Step 5: Establish the weight-value synthesis of reliability Δ*_ij_* by calculating the trusted weight-value *R_j_* of each node using Formulas (12) and (13) in Section 4.1.

Step 6: Calculate the *k* iteration time synchronization conformance vector of node i for the neighbor node *N_i_* , $P\left(k\right)=\left[P_{1}\left(k\right),P_{2}\left(k\right),\ldots ,P_{deg(i)}\left(k\right)\right]$.

Step 7: Calculate the estimated mean value ${\overset{⌣}{P}}_{i}$ of comprehensive time synchronization conformance according to Formula (17). 

Step 8: Estimate the control ability of node *i* for the access transmission of its neighbor node according to its behavior reliability degree (Formula (19)) as mentioned in Section 4.2.

## 5. Performance Analysis and Experimental Results

**Figure 3:**
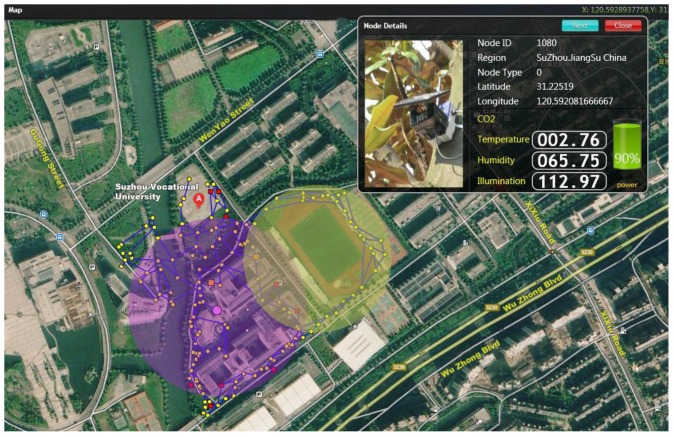
A prototype network for event detection.

This paper evaluates the access performance of the model for event test, taking the wireless sensor network of 200 nodes distributed in the 500 m × 400 m campus of Suzhou Vocational University (shown in Figure 3) as an example. The average communication radius of the node is 10 m. The broadcast period is set to 1 s, the error of the crystal oscillator is generally between 10 ppm and 100 ppm, there is an error of 10 microseconds to 100 microseconds per broadcast period. The he hardware clock skew is randomly selected within the [0.9999,1.0000] interval and the hardware clock offset is randomly selected in the [0,0.0002] interval. The schemes have been evaluated through a real outdoor campus consisting of 150 TelosB motes which run CTP protocol, where CTP is a data collection protocol that dynamically selects the best route to the sink according to a hybrid link estimation algorithm [37]. The operating system is based on TinyOS 2.1.2. The red nodes indicate malicious node in Figure 3. The evaluation will compare three schemes: CEACM-TS, APAC [9] and GTMS (Group based trust management scheme) [8], to show that CEACM-TS is a practical design for the tradeoff between keeping out of the interference and transmitting concurrently, comparing their advantages and disadvantages in terms of random packet losses, error packets, data tampering and retransmission, falsification of identity, denial of service, node capture resetting as well as system cost, energy consumption evaluation and error rate of access control test under the condition of simulative human disturbances and attacks. This topology structure is composed of two backbone networks which contains the cluster head node and sensor nodes. The cluster head node and sensor nodes constitute the multicast tree supported by the backbone network. The cluster head can perform topology control, access control, routing and monitoring the time synchronization state of sensor nodes. Cluster head node stores multicast routing state information, which can minimize routing complexity during link failure. The sensor nodes take part in CEACM-TS algorithm supported by backbone network, saves energy consumption of WSN and improves the performance of the whole network.

During the testing process, the authors set a fixed delay of node information exchange at υ=0.01 s, random delay χ=0.013 s, the number of nodes with no disturbance events occurring in their neighbor node γ=170 and the error probability rate of links of any two nodes ϑ=0.02. As shown in Figure 1, thresholds of two different disturbance areas were set as ψ∈[0.05,0.1] and ψ∈[0.2,0.5].

### 5.1. System Cost

**Figure 4:**
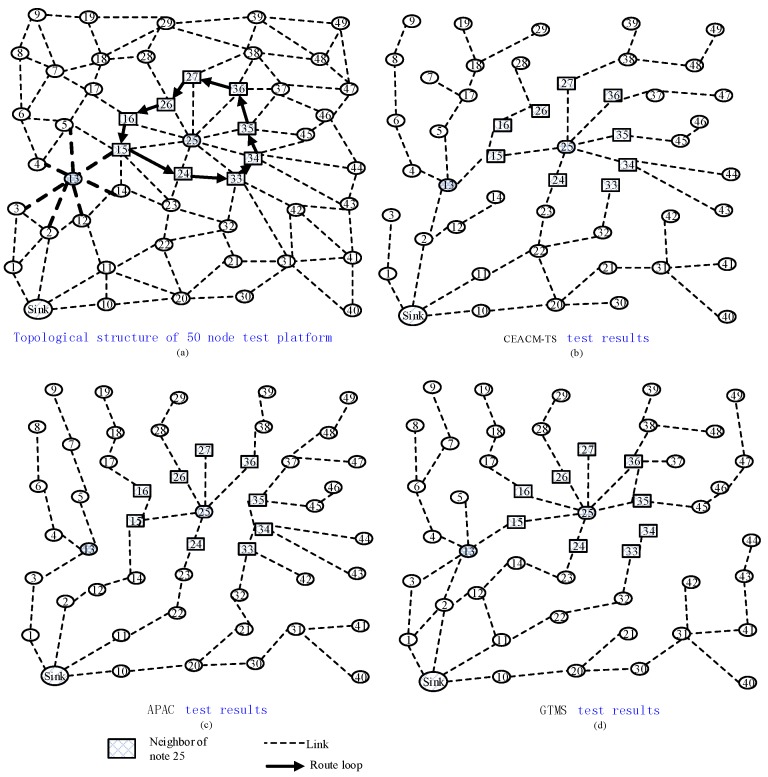
The topological structure of test platform with 50 nodes. (a) Topological structure of 50 node test platform; (b) CEACM-TS test results; (3) APAC test results; (d) GTMS test results.

This test took the network area with a threshold of ψ∈[0.2,0.5], shown in Figure 4, as an example. There were two disturbance factors which caused failure in synchronization. The first was a data jam and missing caused by data tampering and retransmission in node 13. The data packet forwarded by node 13 had to be forwarded from node 15 by a detour. Second, a routing loop (solid line) was formed by nodes capturing resetting in the neighbor nodes of node 25. Thereafter, the authors performed CEACM-TS, APAC and GTMS, respectively, in the network and the network test with collection tree protocol. Test results showed that the tree structure of node 13 and node 25 changed. Both CEACM-TS and APAC broke the routing loop and transmitted data packets through broadcasting RBS protocol packages. When the network topological changed, its neighbor area changed automatically. As the number of neighbors increased, the synchronization and cluster between the depth of topological structures, as well as signal intensity clock drift and clock delay, became more and more close, therefore, the number of the time synchronization proofs required from relevant nodes also increased. 

Figure 4a shows the topology of 50 nodes in our experiments, including some probe traces. The protocol for this network is the CTP, for the analysis of impact with different access control approaches. In this work, we implement three algorithms for access control the network. Figure 4b,c,d describes three convergence tree structures. The process of sampling evidences is used to assign a local time synchronization trust to each node in evidence collection, while establishing convergence tree mainly includes broadcasting and receiving interference noise. Figure 4b shows the probability of a network with a depth less than 3, 4, 5 and 6, formed by CEACM-TS, was respectively 10%, 19%, 29% and 33%. Figure 4c shows the probability of a network with a depth less than 3, 4, 5 and 6 formed by APAC was respectively 8%, 15%, 24% and 27%, Figure 4d shows the corresponding result for GTMS was 25%, 20%, 6% and 6%, respectively. CEACM-TS also greatly reduced the maintenance cost of the time synchronization tree, clock drift and clock delay calculation, compared to APAC and GTMS.

**Figure 5:**
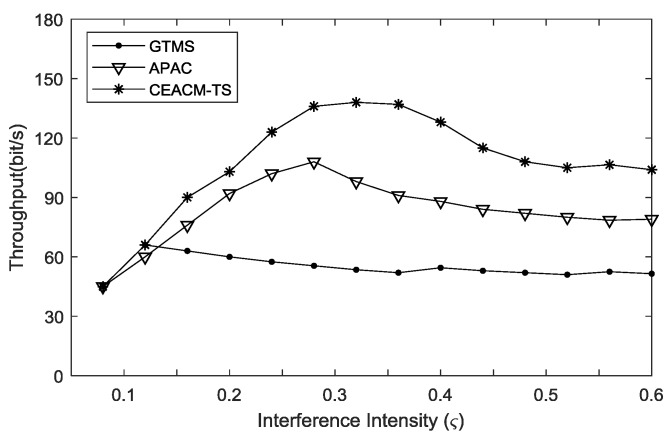
Throughput curve versus Interference intensity.

Figure 5 demonstrates that the three algorithms had similar throughput capacities which increased as the intensity of disturbance noise rose. When the increment of disturbance noise intensity reached a certain threshold, the node gradually became unable to evacuate the caching queue of itself and the throughput capacities of CEACM-TS and APAC decreased. However, when the disturbance intensity was lower than 0.27, the throughput capacities of CEACM-TS and APAC remained increasing with the increment of load strength. The throughput capacity of CEACM-TS was much higher than that of APAC. Both CEACM-TS and APAC performed much better than GTMS in terms of throughput ability. Figure 5 shows that, with further increment of disturbance intensity, the throughput capacity of APAC decreased quickly. When the load intensity was higher than 0.36, the throughput of CEACM-TS was nearly 40 percent at its best performance, which still performed better than GTMS and APAC.

### 5.2. Energy Consumption Evaluation

**Figure 6:**
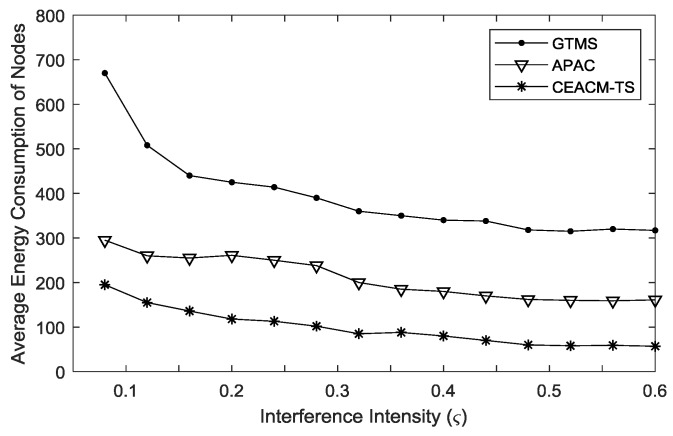
Energy consumption of different interference intensities.

The average energy consumption is defined as the ratio of the total energy cost over the total number of nodes. Figure 6 shows the relation between the average energy consumption and the load intensity. Since the number of data packets sent out by each source node was fixed in this experiment, when the load intensity was low, the network needed more operation cycles to finish the transmission of all data packets. When there was no data packet needing to be transmitted, the idle interception caused energy consumption. The operation cycle was where idle interception occurs. When the increment of load intensity decreased the work efficiency increased, so the average energy consumption generally decreased, as shown in Figure 6. 

Figure 6 shows that the energy consumption of CEACM-TS was lowest among the three algorithms (CEACM-TS, APAC and GTMS). Between APAC and GTMS, APAC performed much better. This is because APAC enabled a data packet to realize multiple hop transmissions in a single cycle, thus, the data packet could be transmitted faster, shortening the working period for transmission of all data packets and saving energy. Due to adopting neighbor invitations and bilinear computer mechanisms, CEACM-TS made full use of sample values in a certain period, which realized swift and even transmission of the caching data packets to the gathering node. When the load intensity was higher than 0.36, CEACM-TS saved energy by 58% and 37% respectively, compared to GTMS and APAC.

### 5.3. Error Rate of Access Control Detection

**Figure 7:**
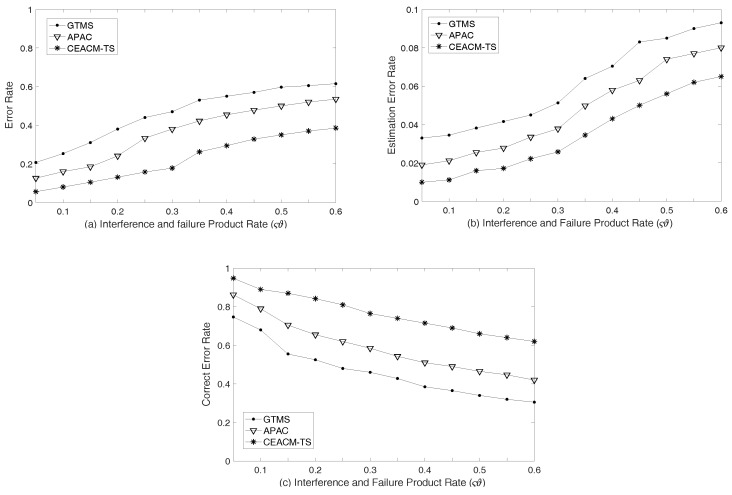
Error rate of access control detection: (**a**) Error rate; (**b**) Estimation error rate; (**c**) Correct error rate.

Figure 7a shows that CEACM-TS was always superior to APAC and GTMS. It had the lowest error rate under different malfunction product rates of node disturbance event ςϑ. When the malfunction product rate of node disturbance event ςϑ was 30%, APAC and GTMS respectively had 38% and 47% disturbance nodes which were not detected, while CEACM-TS only had approximately 15%. Figure 7b shows the probability of conformance estimation error in WSN, with the increment of node disturbance malfunction product rate and the conformance estimates errors of all three algorithms. However, the conformance estimate errors of CEACM-TS was much less than APAC and GTMS. The authors also show the average error correction probability in Figure 7c which demonstrates that, when the node disturbance malfunction product rate was 25%, the error correction probability of APAC and GTMS reached around 62% and 48% respectively, while CEACM-TS reached about 81%, which is significantly higher than that of APAC and GTMS. (Note that the higher percentage is preferred).

Differing from the above analysis is that the introduction of behavior reliability can improve the quality of disturbance event test and lower the effects from disturbance noise, which results in the improvement of reliability and the safety of data transmission. However, due to the impacts on the error tolerance detection from neighbor node disturbance, it is necessary to consider both factors of the node reliability and the number of disturbance neighbor nodes. It can be seen from the above analysis that the introduction of node bilinear behavior reliability can improve the safety of data transmission.

## 6. Conclusions

To improve the safety and fairness in the access control of WSN and, in consideration of the limitations of WSN resources, this paper proposed a WSN access control model CEACM-TS, based on time synchronization with the adoption of a light weight principle and a thought regarding testing time synchronization disturbance noise of the neighbor nodes as the reliability evaluation factor. This distributed access control model realizes reliable access control through two factors: one is the time synchronization similarity between sensor nodes and local sampling of neighbor nodes and the other is the conformity of random statistical characteristics for disturbance behaviors. An algorithm of adaptive weighted data fusion of neighborhood time synchronization is proposed, adaptive optimal weighted value is produced based on measurement interference noise variance estimated and constructed a judgment evidence, the sensor data is adaptive, fast, reasonable grouping weighted, a robust and exact results can be achieved. The method is a simple, practical algorithm and greatly reduces the redundancy of data within the network, saving a lot of storage resources and network bandwidth. This model can efficiently solve the problems faced by internal reliability and the external safety of data transmission due to its characteristics in distribution, low complexity, strong extendibility and robustness. Since the model has a strong extendibility, it still can work when the network scale changes. Furthermore, the model only requires time synchronization communication between nodes and neighbor nodes to reach the overall target. This avoids energy consumption caused by multi-hop communication and reduces the demand for storage space. Current test results showed the applications of the model in WSN mainly included sensor fusion and filtration, time synchronization, target location and tracing, sensor scheduling and more, which improved the ability of nodes in controlling neighbor access transmission and virtually reduced illegal access between neighbor nodes.
